# Activation of hepatic acetyl-CoA carboxylase by S-nitrosylation in response to diet

**DOI:** 10.1016/j.jlr.2024.100542

**Published:** 2024-04-17

**Authors:** Nicholas M. Venetos, Colin T. Stomberski, Zhaoxia Qian, Richard T. Premont, Jonathan S. Stamler

**Affiliations:** 1 Department of Medicine, Institute for Transformative Molecular Medicine, Case Western Reserve University, Cleveland, OH, USA; 2 Harrington Discovery Institute, University Hospitals Cleveland Medical Center, Cleveland, OH, USA

**Keywords:** cell signaling, liver, dietary fat, triglycerides, S-nitrosylation, acetyl-CoA carboxylase, lipogenesis

## Abstract

Nitric oxide (NO), produced primarily by nitric oxide synthase enzymes, is known to influence energy metabolism by stimulating fat uptake and oxidation. The effects of NO on de novo lipogenesis (DNL), however, are less clear. Here we demonstrate that hepatic expression of endothelial nitric oxide synthase is reduced following prolonged administration of a hypercaloric high-fat diet. This results in marked reduction in the amount of S-nitrosylation of liver proteins including notably acetyl-CoA carboxylase (ACC), the rate-limiting enzyme in DNL. We further show that ACC S-nitrosylation markedly increases enzymatic activity. Diminished endothelial nitric oxide synthase expression and ACC S-nitrosylation may thus represent a physiological adaptation to caloric excess by constraining lipogenesis. Our findings demonstrate that S-nitrosylation of liver proteins is subject to dietary control and suggest that DNL is coupled to dietary and metabolic conditions through ACC S-nitrosylation.

Acetyl-CoA carboxylase (ACC) catalyzes the carboxylation of cytosolic acetyl-CoA into malonyl-CoA and is the rate-limiting step of the de novo lipogenesis (DNL) synthetic pathway that produces endogenous fatty acids (1). Although the major site of DNL is the liver, it functions to a lesser extent in other tissues including adipose tissue (2). Proper regulation of DNL is critical for maintenance of metabolic homeostasis, and its dysregulation is implicated in a host of metabolic disorders, including insulin resistance, obesity, and hepatic steatosis (2). As is typical of key metabolic enzymes, ACC is subject to extensive regulation, especially in response to dietary conditions. The transcription factors SREBP1 and ChREBP increase ACC expression in response to insulin or carbohydrates, respectively (1). ACC is activated allosterically by citrate and feedback-inhibited by acyl-CoAs (1). AMPK inhibits ACC via phosphorylation when cellular energy levels are low (1). The net effect of this multifaceted regulation is enhanced DNL during carbohydrate and energy excess and inhibition of the pathway during periods of energy scarcity.

Nitric oxide (NO), produced by nitric oxide synthases (NOSs), is a well-known modulator of energy metabolism and metabolic health (3, 4). The precise nature of NO’s effect on metabolism is heavily context dependent, where source, location, and target give rise to different functional outcomes (3, 4). Much of this complexity likely arises from regulated S-nitrosylation, the posttranslational modification of protein cysteines by NO (5). Accumulating evidence suggests S-nitrosylation is carried out by nitrosylase and denitrosylase enzymes that draw mechanistic analogy to acetyltransferase and deacetylases (6). However, while acetylation plays a recognized role in metabolic regulation, the effects of S-nitrosylation are less clear. As one example, the terminal enzyme in DNL, FASN, is subject to stimulatory S-nitrosylation in differentiating adipocytes to facilitate lipid accumulation (7). But DNL is regulated primarily at the level of ACC (1). It is therefore of interest that S-nitrosylated ACC (8, 9) has been detected in untargeted proteomic screens.

In the present study, we identify endogenous S-nitrosylation of ACC in hepatocytes and demonstrate marked enzymatic activation by this modification. We further show that the degree of hepatic ACC S-nitrosylation is coupled to dietary constituents. This work identifies S-nitrosylation as a novel and dynamic mechanism for modifying ACC activity, adding to its existing regulatory framework.

## Materials and methods

### Animals

Animal experiments were approved by the Case Western Reserve University Institutional Animal Care and Use Committee and complied with the Guide for the Care and Use of Laboratory Animals and the American Veterinary Medical Association guidelines on euthanasia. Two-month-old mice were fed the following from Research Diets, Inc.: AIN-93M Mature Rodent Diet (D10012M, referred to as standard diet, SD) or Rodent Diet with 60 kcal% Fat (D12492, referred to as high-fat diet, HFD). SD contained ∼3.9 kcal per gram and had the following composition: 15% kcal protein, 76% kcal carbohydrate, and 9% kcal fat. HFD contained ∼5.2 kcal per gram and had the following composition: 20% kcal protein, 20% kcal carbohydrate, and 60% kcal fat.

Primary hepatocytes were isolated from mice as described previously (10) by perfusion with collagenase, plated onto collagen-coated plates, and maintained in William’s Medium E (Sigma, W1878) containing 10% FBS, 1% Pen-strep, and Primary Hepatocyte Maintenance Supplement (Thermo, CM4000).

### Partial purification of ACC

ACC was prepared from mouse liver as described previously (11, 12), with minor modifications. Briefly, livers were removed from mice and washed in ice-cold PBS prior to homogenization in 5 volumes homogenization buffer (50 mM potassium phosphate, pH 7.5, 10 mM EDTA, 10 mM 2-mercaptoethanol, Roche protease inhibitors, and Roche phosphatase inhibitors) in a prechilled Dounce homogenizer. The homogenate was made 3% polyethylene glycol (PEG) using a 50% PEG solution and centrifuged at 20,000 *g* for 15 min. The supernatant was adjusted to 5% PEG, mixed for 5 min at 4°C, and centrifuged at 20,000 *g* for 20 min. The pellet was washed with ice-cold H_2_O and resuspended in 25% of the original homogenization buffer volume. Ammonium sulfate (200 mg/ml) was added while stirring for 45 min at 4°C. Following centrifugation at 20,000 *g* for 30 min, the pellet was resuspended in an equal volume of resuspension buffer (20 mM Hepes, pH 7.6, 150 mM NaCl, 1 mM EDTA, 1 mM EDTA, 10% glycerol, and Roche protease inhibitors).

### Western blot analysis

Western blot analysis of protein expression was performed according to standard procedures. The following primary antibodies were used: ACC (Cell Signaling, 3676, which recognizes both ACC1 and ACC2), endothelial nitric oxide synthase (eNOS) (Cell Signaling, 32027), inducible nitric oxide synthase (iNOS) (Cell Signaling, 13120), neuronal NOS (Cell Signaling, 4231), GAPDH (Proteintech, 10494-1-AP), and P97 (Fitzgerald, 10R-P104A).

### Resin-assisted capture of S-nitrosylated protein analysis

For analysis of S-nitrosylated proteins (SNO-proteins) in tissue and cells, samples were lysed in HEPES-EDTA-Neocuproine buffer (HEN) buffer, pH 7.8 (100 mM Hepes, 1 mM EDTA, 0.1 mM neocuproine) containing 150 mM NaCl, 1% Nonidet P-40, 0.01% S-methyl methanethiosulfonate (MMTS), and protease inhibitors. Following centrifugation, SDS (2.5% final) and MMTS (0.2% final) were added to the supernatant. Samples were incubated in a water bath at 50°C for 20 min. Proteins were precipitated with ice-cold acetone and re-dissolved in HEN buffer with 1% SDS (HENS). Precipitation and resuspension were repeated, and equal amounts of protein per sample were combined with thiopropyl-Sepharose (GE) and incubated in the presence or absence of 30 mM ascorbate for <4 h with rotation away from light. Protein-bound beads were washed four times with HENS buffer and twice with HENS buffer diluted 1:10 with H_2_O. SNO-proteins were eluted in 2× loading dye containing 10% 2-mercaptoethanol and used for SDS-PAGE. Western blot analysis was used to detect specific proteins, and Imperial Protein Stain (Thermo, 24615) was used to detect total protein.

For analysis of SNO-ACC following partial purification, SNO-donors were prepared by combining equal volumes of 100 mM sodium nitrite with 100 mM acidified Coenzyme A (CoA) or L-cysteine. SNO-donors were incubated with 50–200 μg prepared ACC for 10 min at room temperature before the addition of HENS containing 2.5% SDS, MMTS (0.2% final), and 2 mg BSA. Resin-assisted capture of S-nitrosylated proteins (SNO-RAC) proceeded as described above.

### Quantitative real-time PCR analysis

For RT-qPCR analysis, mRNA was isolated from livers using the RNeasy Lipid Tissue Mini Kit (Qiagen, 74804) following manufacturer’s instructions. RNA was converted to cDNA using High-Capacity RNA-to-cDNA kit (Thermo, 4387406). Quantitative PCR was performed on the Applied Biosystems StepOnePlus Real Time PCR System using TaqMan Fast Advanced Master Mix, according to manufacturer’s protocols. Data were analyzed using the ΔΔCT method. The following TaqMan Assay probes were used: *Nos2* (Thermo, Mm00440502_m1), *Nos3* (Thermo, Mm00435217_m1), and *Ppia* (Thermo, Mm02342430_g1). *Ppia* was used as an internal control.

### Radiometric ACC activity assay

ACC activity was measured as described previously (12), with minor modifications. Each reaction containing 55 μg ACC, 10 mM MgCl_2_, and 1 mg/ml BSA in 40 mM Hepes (pH 7.6) was incubated at a final volume of 100 μl for 10 min at room temperature with or without citrate (10 mM final) and the indicated concentrations of NO donor (prepared as described above). Following incubation, an equal volume of reaction buffer (40 mM Hepes, pH 7.6, 10 mM MgCl_2_, 5 mM ATP, 25 mM NaHCO_3_, 1.5 μCi NaH^14^CO_3_) with or without 0.284 mM acetyl-CoA was added, and reactions were incubated at room temperature for 1.5 h. Reactions were terminated by the addition of 60 μl 6N HCl, transferred to liquid scintillation vials, and evaporated to dryness. Liquid scintillation cocktail was added, and [^14^C] radioactivity was quantified.

### Statistical analysis

Statistical analysis was performed using GraphPad Prism 10. Unpaired *t* tests and one-way ANOVA were used as indicated. Appropriate multiple comparisons testing was performed as indicated. *P* values <0.05 were considered statistically significant, and actual values are displayed in figures.

## Results

### Hepatic eNOS is regulated by metabolic and dietary conditions

Reversible S-nitrosylation allows for dynamic regulation of protein function in response to external stimuli (5). eNOS is the primary source of NO in the liver (13). In rats, short-term high-fat feeding results in downregulation of eNOS expression and reduced NO bioavailability by 1 week (14). Therefore, we hypothesized that high-fat feeding should decrease S-nitrosylation of proteins to facilitate metabolic adaptation to a hypercaloric diet.

**Fig. 1:**
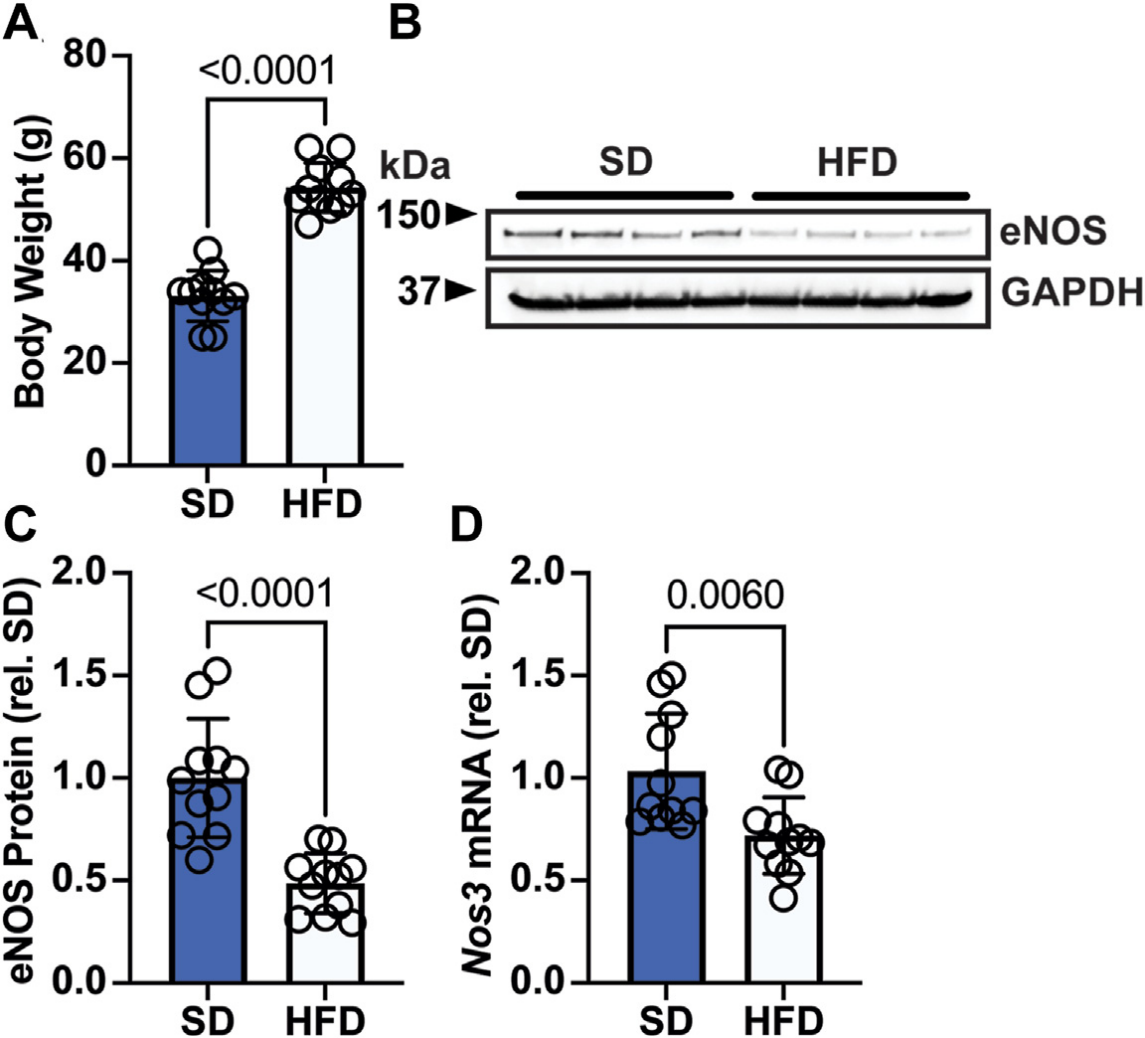
Hypercaloric high-fat diet alters liver eNOS expression. A: Body weight in grams (g) of mice following 4 months of standard diet (SD) or high-fat diet (HFD) feeding; n = 11 per group. B: Representative Western blot analysis of eNOS protein expression in livers from mice following 4 months of SD or HFD feeding. C: Quantification of *B*, n = 11 per group. D: Quantitative real-time PCR analysis of *Nos3* mRNA in livers from mice following 4 months of SD or HFD feeding; n = 11 per group. Data represent mean ± SD. *P* value was calculated by unpaired *t* test. eNOS, endothelial nitric oxide synthase.

**Supplemental Fig. S1:**
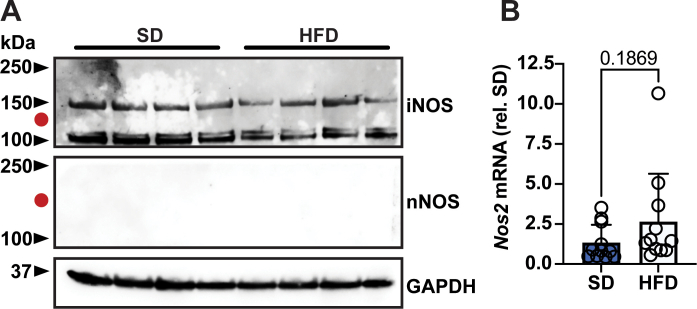
Hypercaloric high-fat diet does not affect liver iNOS or nNOS expression. A: Western blot analysis of inducible nitric oxide synthase (iNOS) and neuronal nitric oxide synthase (nNOS) protein expression in livers from mice following 4 months of standard diet (SD) or high-fat diet (HFD) feeding. Arrows on left indicate molecule weight markers in kDa. No signal was observed for iNOS or nNOS at the predicted molecular weights (130 kDa and 160 kDa, respectively, red dots). GAPDH is used as an internal control. B: Quantitative real-time PCR analysis of *Nos2* mRNA in livers from mice following 4 months of SD or HFD feeding; n = 11 per group. Data represent mean ± SD. *p* value was calculated by unpaired *t* test

To test this hypothesis, we subjected 2-month-old wild-type mice to either the standard chow diet (SD) or a hypercaloric HFD. This HFD contained 60% kcal fat and 20% kcal carbohydrate, relative to 9% kcal fat and 76% kcal carbohydrate in SD, and had 33% more calories per gram. Following 4 months of feeding, HFD-fed mice demonstrated markedly increased body weight (Fig. 1A). Importantly, HFD-fed mice had significantly decreased levels of hepatic eNOS mRNA and protein compared to the SD mice (Fig. 1B–D). Neither neuronal NOS nor iNOS protein were observed in liver to any appreciable degree under either condition (supplemental Fig. S1A) and quantitative real-time PCR analysis showed no differences in iNOS mRNA between groups (supplemental Fig. S1B).

### Liver protein S-nitrosylation is subject to dietary regulation

**Fig. 2:**
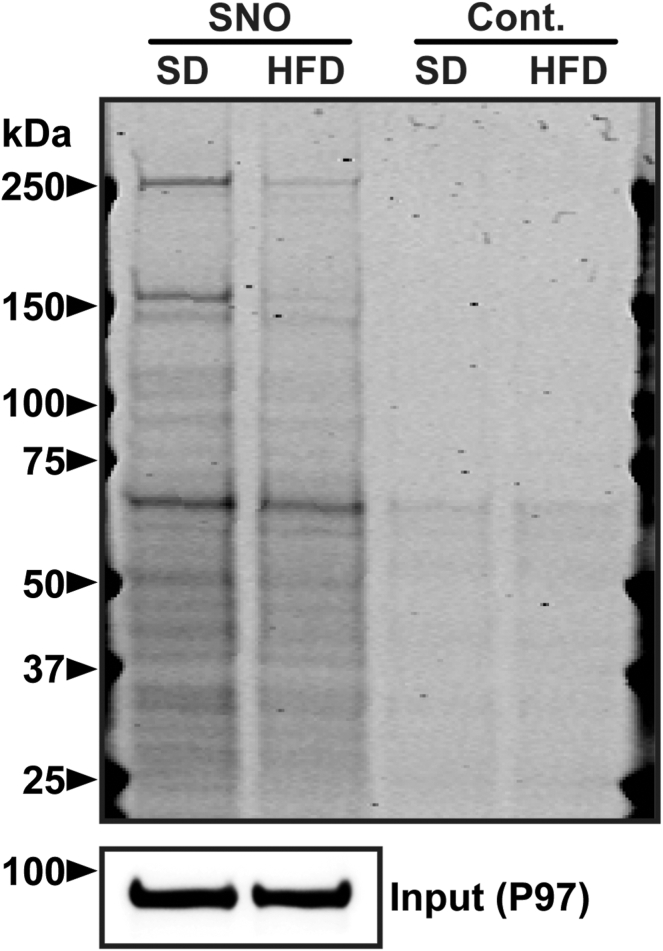
Protein S-nitrosylation. Global amounts of SNO-proteins in livers from mice following 4 months of SD or HFD feeding. SNO-RAC eluate was separated by SDS-PAGE and stained with Imperial blue. Image is representative of three replicates. Control (Cont.) lanes represent resin-assisted capture of the corresponding samples in the absence of ascorbate to demonstrate specificity for SNO modification. SNO-proteins, S-nitrosylated proteins.

**Supplemental Fig. S2:**
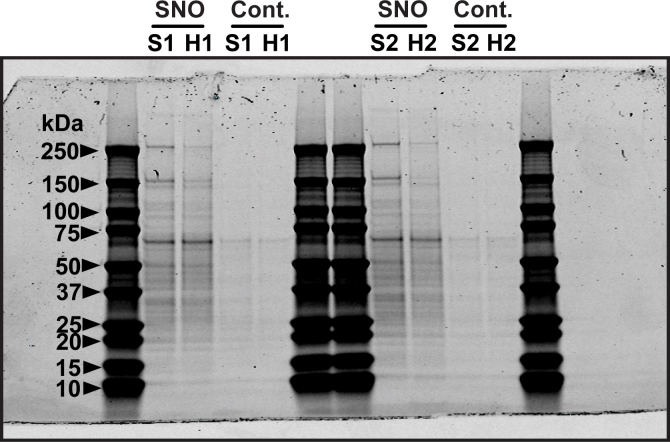
Protein S-nitrosylation. Global amounts of SNO-proteins in livers from mice following 4 months of SD or HFD feeding. Uncropped, full resolution image of Fig. 2. Two biological replicates are shown. Control (Cont.) lanes represent resin assisted capture of the corresponding samples in the absence of ascorbate to demonstrate specificity for SNO modification

Based on the above data, we tested whether hepatic eNOS expression might be associated with concordant changes in protein S-nitrosylation. We employed SNO-RAC followed by SDS-PAGE and Coomassie staining of the S-nitrosylated fraction of total protein. This analysis revealed a decline in overall protein S-nitrosylation in the HFD liver compared to SD liver, especially of high molecular weight SNO-protein species >∼70 kDa (Fig. 2, and supplemental Fig. S2). In particular, major bands at ∼150 kDa and >250 kDa are substantially reduced in HFD liver (Fig. 2, and supplemental Fig. S2).

### ACC is subject to S-nitrosylation

**Fig. 3:**
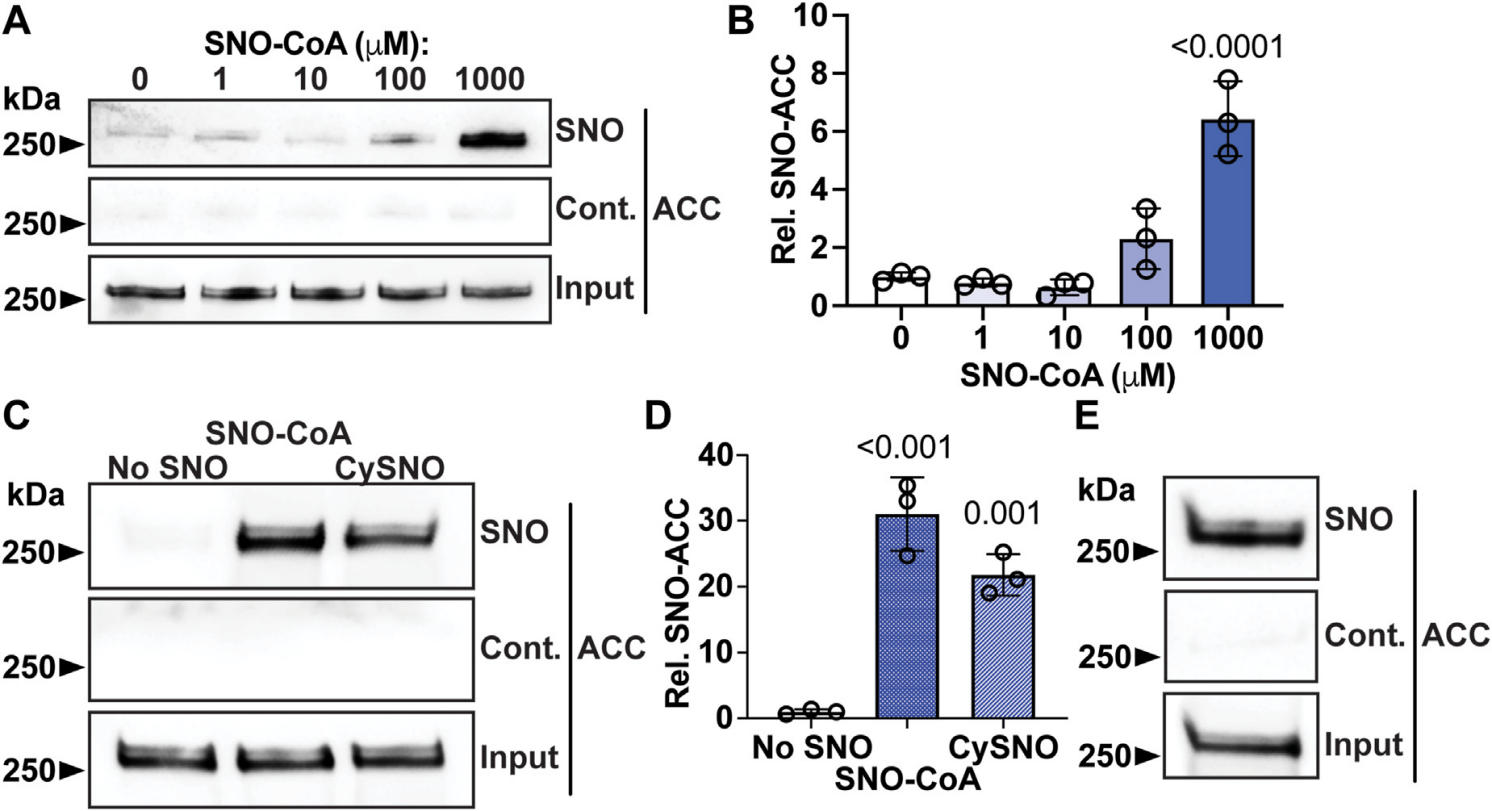
ACC is S-nitrosylated. A: S-nitrosylation of partially purified acetyl-CoA carboxylase (ACC) (SNO-ACC) from mouse liver following incubation with various concentrations of S-nitroso-coenzyme A (SNO-CoA). A control without ascorbate (Cont.) is included to confirm specificity of S-nitrosylation. B: Quantification of *A*; n = 3. C: S-nitrosylation of partially purified ACC (SNO) from mouse liver following incubation with 1 mM SNO-CoA or S-nitrosocysteine (CySNO). D: Quantification of (C); n = 3. E: Endogenous S-nitrosylation of ACC in primary mouse hepatocytes. Data represent mean ± SD. *P* value was calculated by one-way ANOVA with Tukey’s correction and indicate comparison to groups without SNO-donor.

**Supplemental Fig. S3:**
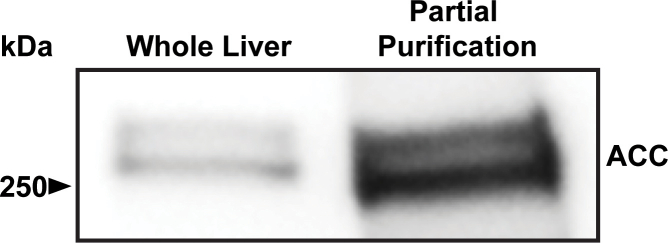
Partial purification of liver ACC. Western blot analysis of ACC protein expression in whole liver lysate or after enrichment by partial purification. Equal amounts of protein were loaded for both groups

DNL, the primary metabolic pathway for fat synthesis, is regulated by dietary fat content (15, 16), and its rate-limiting enzyme is ACC. ACC is highly expressed in liver and has two isoforms encoded by distinct genes (ACC1 by *Acaca* and ACC2 by *Acacb*) with molecular weights ∼260–270 kDa; both isoforms have been identified as putative SNO-proteins in proteomic screens (8, 9, 17). We therefore hypothesized that ACC may be S-nitrosylated in liver and may be among the SNO-proteins altered by dietary fat and calorie content. To validate ACC S-nitrosylation, we first purified ACC from mouse liver, which appears as two bands presumably representing ACC1 and ACC2 (supplemental Fig. S3). Incubation of purified ACC with the metabolic cofactor (18) SNO-CoA produced significant increases in S-nitrosylation of both ACC bands (Fig. 3A–C), as assessed by SNO-RAC analysis. S-nitrosylation of ACC was similarly observed following treatment with NO donor S-nitrosocysteine (CySNO) (Fig. 3C, D). Finally, endogenous S-nitrosylation of ACC was observed in primary hepatocytes isolated from untreated wild-type mice (Fig. 3E).

**Fig. 4:**
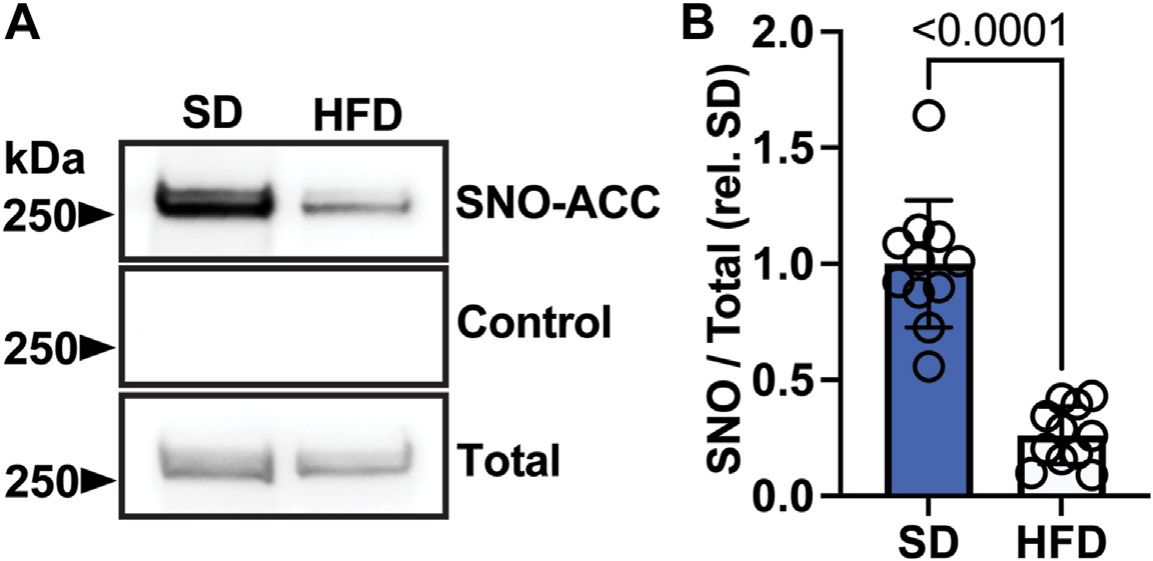
ACC S-nitrosylation in vivo changes after HFD. A: S-nitrosylation of ACC (SNO) in livers from mice following 4 months of SD or HFD feeding. B: Quantification of (B); n = 11 per group. Data represent mean ± SD. *P* value was calculated by unpaired *t* test.

**Supplemental Fig. S4:**
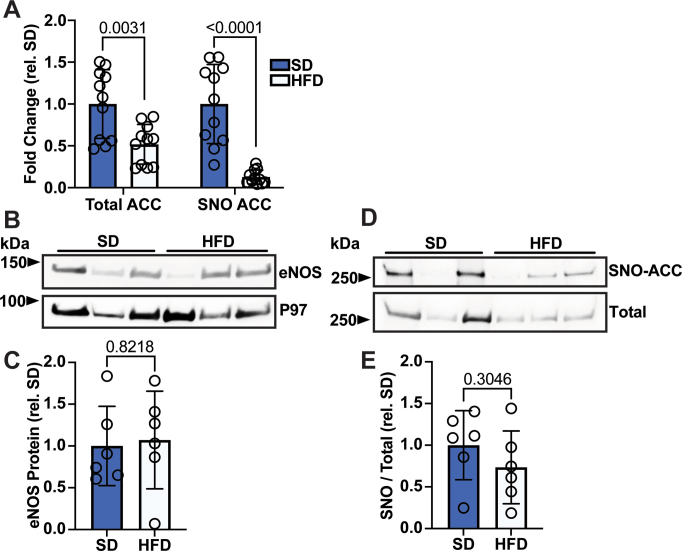
ACC S-nitrosylation in vivo changes after HFD. A: Fold change in total ACC or endogenously S-nitrosylated (SNO) ACC in livers from mice following 4 months of SD or HFD feeding. Expression levels are normalized to SD for both total and SNO. B: Representative Western blot analysis of eNOS protein expression in white adipose tissue from mice following 4 months of SD or HFD feeding. C: Quantification of (B), n = 6 per group. D: S-nitrosylation of ACC (SNO) in white adipose tissue from mice following 4 months of SD or HFD feeding. E: Quantification of *D*; n = 6 per group. Data represent mean ± SD. *p* value was calculated by individual unpaired *t* test

Importantly, HFD feeding triggered a notable drop in S-nitrosylation of both ACC bands in whole liver (Fig. 4A, B). It has been reported that dietary fat reduces DNL (15, 19) through declines in ACC expression. However, lower expression of ACC in HFD liver could only account for ∼50% of the reduction in SNO-ACC (supplemental Fig. S3A). To investigate the specificity of these hepatic adaptations to prolonged hypercaloric diet, we also measured eNOS expression and SNO-ACC in white adipose tissue from SD and HFD mice, observing no differences in either (supplemental Fig. S4B–E). Thus, the effects of diet on ACC S-nitrosylation are specific to liver.

### S-nitrosylation enhances ACC enzymatic activity

**Fig. 5:**
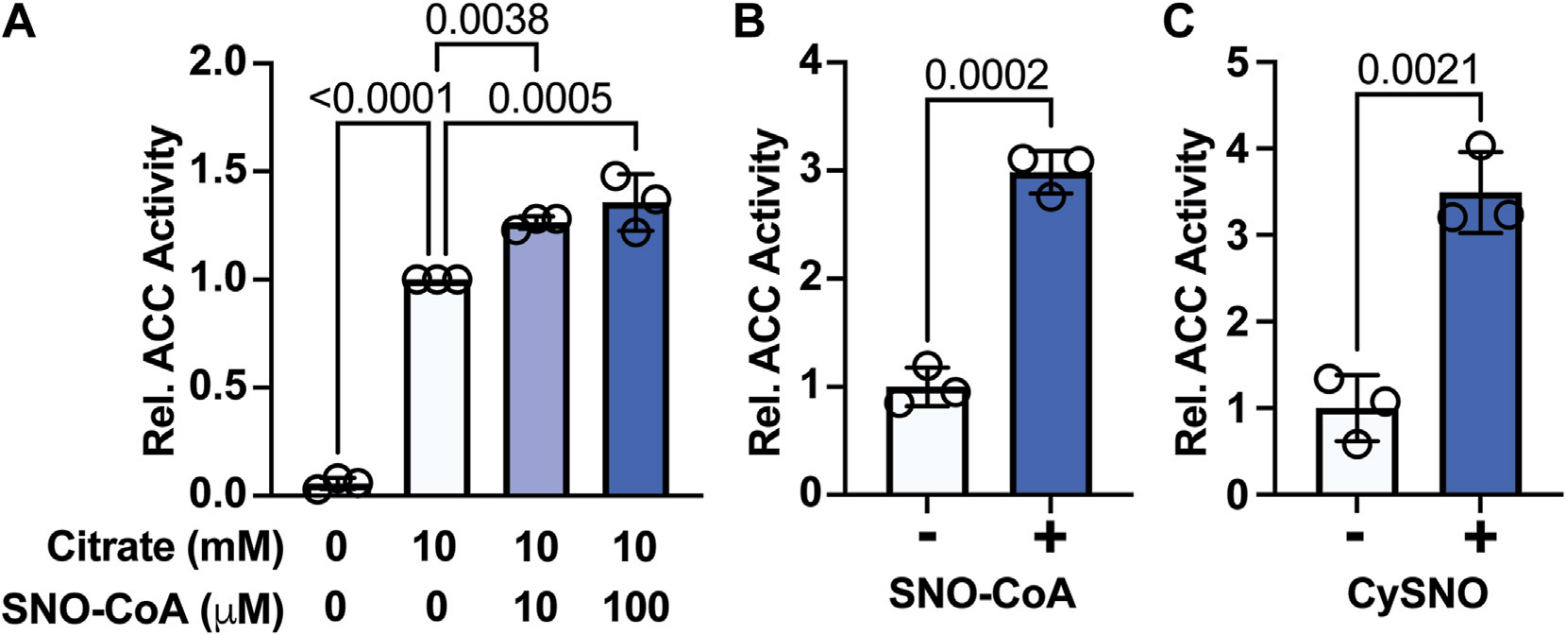
ACC S-nitrosylation increases enzyme activity. A: Enzymatic activity of partially purified ACC from mouse liver following incubation with various concentrations (0, 10, and 100 μM) of SNO-CoA in the presence or absence of 10 mM citrate. Data are normalized to 10 mM citrate without SNO-CoA within each replicate. B, C: Enzymatic activity of partially purified ACC from mouse liver following incubation with (+) or without (−) 1 mM SNO-CoA (B) or CySNO (C) in the presence of 10 mM citrate. Data represent mean ± SD. A: *P* value was calculated comparing all groups to 10 mM citrate without SNO-CoA by one-way ANOVA with Dunnett’s multiple comparisons test. B, C: *P* value was calculated by unpaired *t* test.

We next sought to determine the functional effect of ACC S-nitrosylation. We performed radiometric activity assays with ACC preparations from liver in the presence of increasing concentrations of SNO-CoA. Purified ACC alone had very low activity (Fig. 5A), so we measured ACC activity in the presence of citrate as a stimulatory cofactor. In accordance with previous studies (12, 20, 21), citrate dramatically increased ACC activity (Fig. 5A). In the presence of citrate, low micromolar to millimolar SNO-CoA further enhanced ACC activity by up to ∼300% (Fig. 5A, B).

To confirm that ACC activity was enhanced specifically by S-nitrosylation rather than by binding of the CoA cofactor (potentially derived from added SNO-CoA), we repeated assays using CySNO. In the presence of citrate, CySNO led to a >300% induction of ACC activity (Fig. 5C). Collectively, these data suggest that S-nitrosylation activates ACC (in stark contrast to phosphorylation, which inhibits ACC activity (1)). In a physiological context, HFD-induced eNOS downregulation and consequent ACC denitrosylation helps reduce endogenous fat synthesis in lipid-laden hepatocytes where exogenous fats are plentiful. Conversely, in SD conditions, elevated eNOS expression and S-nitrosylation of ACC would facilitate DNL flux when fats are needed.

## Discussion

We demonstrate that protein S-nitrosylation in the liver is coupled to dietary fat calorie intake and metabolic health. We further show that S-nitrosylation of ACC, which markedly activates the enzyme, is regulated by diet. Specifically, a hypercaloric diet rich in fat reduces S-nitrosylation of ACC because of diminished eNOS activity, thereby likely counteracting hepatic lipid deposition. These adaptations are specific to liver where excessive fat deposition can be injurious (supplemental Fig. S4B–E). Collectively, our data provide a physiological basis for regulation of ACC by S-nitrosylation and for decreases in eNOS activity in states of high caloric intake.

Although an exact mechanism for reduced eNOS expression in our study is not known, it may represent a downstream consequence of obese or steatotic states. Indeed, obesity triggers increased circulating levels of the adipokine TNFα (22), while hepatic steatosis provokes local hypoxia (23). Both TNFα and chronic hypoxia are negative regulators of eNOS transcription and mRNA stability (24), and we observe significant reductions in eNOS mRNA following prolonged hypercaloric HFD feeding (Fig. 1). Notably, hypercaloric HFD, as used in our study, reduces eNOS expression in rodents (14), whereas isocaloric HFD does not (25). This suggests that dietary composition alone may not be sufficient to regulate eNOS expression, in keeping with the idea that additional metabolic disturbances (obesity, inflammation, and hypoxia) play contributing roles.

ACCs are large (>250 kDa) proteins consisting of three domains: a biotin carboxylase domain, a biotin carboxyl carrier protein domain, and a carboxyl transferase (CT) domain (1). Following bicarbonate-dependent carboxylation of biotin in the biotin carboxylase domain, the biotin carboxyl carrier protein domain facilitates transfer of the carboxyl group from biotin to acetyl-CoA within the CT domain of a neighboring ACC (1). Two isoforms of ACC exist with distinct but overlapping functions. ACC1 contains 28 cysteines and generates malonyl-CoA for biosynthesis of fatty acids (1). ACC2 contains 42 cysteines and is thought to produce malonyl-CoA to allosterically regulate fatty acid oxidation (1). Interestingly, two separate site-specific SNO-proteomics screens identified the same cysteine within the CT domain (C1768 in mouse (8), C1769 in humans (9)) of ACC1 as a potential SNO-site, along with other residues. Cys86 has also been identified as a putative SNO-site in mouse ACC2 (17). Future studies should determine the mechanistic underpinning of SNO-dependent ACC activation, which is recapitulated in vitro by SNO-CoA, noting the recent discovery of enzymes that catalyze S-nitrosylation utilizing SNO-CoA (18).

Calorically dense diets high in exogenous fats, and the resulting obesity, have been shown to significantly reduce hepatic lipogenesis in rodents (15, 16) while at the same time downregulating AMPK (26). Attenuated AMPK activity would be expected to reduce ACC phosphorylation and therefore activate ACC under these conditions. Our observation that stimulatory ACC S-nitrosylation is reduced by overnutrition may serve to reconcile these opposing observations by counterbalancing or overriding the effects of reduced ACC phosphorylation. Accumulating evidence suggests coregulation of proteins by S-nitrosylation and phosphorylation (β-arrestin2, β_2_-adrenergic receptor, and insulin receptor) (18, 27, 28), and ACC exemplifies such crosstalk. Future studies should determine levels of SNO-ACC and phospho-ACC under different dietary conditions to validate the opposing roles of regulatory modifications in DNL.

Aberrantly elevated lipogenesis is a hallmark of hepatic steatosis (29) and inhibitors of ACC ameliorate steatosis (30). As discussed, reduced SNO-ACC may represent a compensatory physiological response to dietary insult in the liver. By contrast, significant evidence suggests a protective role for hepatic eNOS in diet-induced steatosis (3, 31). As demonstrated in Fig. 3, the S-nitrosylation state of many proteins drops when eNOS levels are reduced. It is therefore likely that reduced S-nitrosylation of other proteins may offset the beneficial effect of reduced SNO-ACC. Overall, impaired eNOS-derived NO bioavailability is known to negatively impact steatosis (3, 31). As one example, eNOS enhances mitochondrial biogenesis and respiration (3), which would directly counter fatty acid synthesis through ACC. Identification of SNO-proteins that are subject to NO regulation during dietary changes may reveal additional insights.

In sum, our results identify a novel mechanism of regulation of ACC, the rate limiting enzyme in DNL. Specifically, we demonstrate that S-nitrosylation of ACC results in a marked increase in enzyme activity. We further show that S-nitrosylation of ACC is both dynamic and responsive to dietary and metabolic inputs. ACC is emerging as a promising therapeutic target in a variety of metabolic disorders (30) and its functional regulation by S-nitrosylation may offer additional therapeutic strategies to affect this important enzyme.

## Data availability

Data used in this study are available in the article and the supplement or from the corresponding author upon request.

## Supplemental data

This article contains supplemental data.

## Conflict of interest

J. S. S. is a founder and board member of and has equity interest in SNO bio, a company developing nitrosylation-related therapeutics, and NNOXX, a company developing NO-based device technology. CWRU and UHCMC are aware of these conflicts and appropriate management plans are in place. None of the other authors have relevant conflicts to disclose.
